# Changes in awareness of condition in people with mild‐to‐moderate dementia: Longitudinal findings from the IDEAL cohort

**DOI:** 10.1002/gps.5702

**Published:** 2022-03-16

**Authors:** Catherine M. Alexander, Anthony Martyr, Linda Clare, in collaboration with the IDEAL Programme Research Team

**Affiliations:** ^1^ REACH: The Centre for Research in Ageing and Cognitive Health, University of Exeter Medical School, University of Exeter, South Cloisters, St Luke's Campus Exeter UK; ^2^ National Institute for Health Research Applied Research Collaboration South‐West Peninsula Exeter UK

**Keywords:** anosognosia, case‐match, co‐morbidity, denial, insight

## Abstract

**Objectives:**

Awareness of difficulties shown by people with dementia is known to vary, but few studies have explored changes in awareness over time. Investigating this could further the understanding of surrounding concepts and reasons for impaired awareness. Recognising emerging or diminishing awareness could facilitate discussions about diagnosis and appropriate post‐diagnostic support.

**Methods:**

Using longitudinal data from the Improving the experience of Dementia and Enhancing Active Life (IDEAL) cohort, awareness in community‐dwelling people with mild‐to‐moderate dementia was assessed at three timepoints over 2 years. A validated checklist was used to evaluate awareness of difficulties associated with dementia. We examined changes in awareness for people with low awareness at baseline, and used case‐matching to describe differences in characteristics between people who gained awareness, and those who continued with low awareness.

**Results:**

At baseline, 83 people from a sample of 917 showed low awareness. The majority of those remaining in the study at later timepoints had gained awareness, some as late as four or more years after diagnosis. Case‐matched comparisons revealed few distinguishing characteristics: cases with stable low awareness had similar or better cognitive and functional ability than those who gained and retained awareness at 12 and 24 months, but may have had more co‐morbidities.

**Conclusions:**

Self‐reported awareness of difficulties can change and may increase over time in people with mild‐to‐moderate dementia. There may be individual reasons for ongoing low awareness, not explained by cognitive or functional ability. This challenges the view that a single record of low awareness represents a fixed disease‐related symptom, and highlights the complex, individual and dynamic nature of awareness.

## Key points

- Awareness of condition varies in people with mild‐to‐moderate dementia.
- Awareness of condition can change over time, with delayed acknowledgement of difficulties in some people.
- Individual factors beyond the stage of disease may influence awareness of difficulties in dementia.

## 1 INTRODUCTION

Receiving a dementia diagnosis has immediate implications for the person diagnosed and their family, with a prognosis of ongoing decline in abilities affecting future independence, relationships, living situation, finances and ultimately, reducing life expectancy.^1^, ^2^ People with dementia do not all acknowledge their condition and some appear to lack awareness of dementia‐related decline in cognition, everyday functioning in activities of daily living, and/or social ability.^3^, ^4^ Impaired awareness, also termed anosognosia, is sometimes viewed as a symptom of dementia, described as lack of insight, or seen as a fixed or progressive entity, but few longitudinal studies have explored whether awareness increases.^5^

Of the few recent studies that investigated change in awareness over time, findings are equivocal, showing decline, stability, or increase in awareness. One of these studies found a high prevalence of anosognosia at baseline (30.9%), increasing to 39.4% with declines overall in levels of awareness at 18 months.^6^ Another reported a similar proportion of people with low awareness at baseline (39.5%), with high rates of persistence (80%) and incidence (38.3%) at 12 months, although some remissions to improved awareness (20%) were noted.^7^ A study with a longer follow‐up period reported a smaller proportion of people with no awareness at baseline (13%), finding overall that 39% people had declined in awareness at 36 months, but 16% had improved and the rest were stable.^8^ An earlier study in 2012 found that awareness was largely stable, with declines in just 6% and improvements in 3% of the participants at 20 months.^9^

However, the differing results are complicated by the different methods used to look at various aspects of awareness. For example, some researchers compared self‐evaluation of a range of abilities with an informant rating,^7^, ^9^ or used clinician ratings of global awareness.^6^, ^8^ The populations studied also varied, with lower cognitive scores at baseline, suggesting more cognitive impairment, in two studies.^6^, ^7^ A review concluded that while awareness reduces with increasing dementia severity, there is variability at any stage.^10^

In dementia, awareness is complex and heterogeneous.^11^, ^12^ Apart from disease‐related changes, the involvement of denial as a subconscious defence, or minimization as a coping strategy, might explain how people adjust differently to a diagnosis of dementia,^13^ and how this may change over time and with support.^14^ The different factors influencing awareness^3^ may contribute to different trajectories of awareness.^9^

For people unable to recognise symptoms or not ready to discuss dementia, untimely information could be confusing or harmful.^15^ Focusing on maintaining the status quo may assist coping^16^; lack of awareness of future decline may help preserve quality of life (QoL),^17^ and being less aware of dementia symptoms may sustain better mood and greater perceived ability to ‘live well’.^18^ However, support needs may change if individuals become more aware of their condition. Understanding changes in awareness could help disentangle the mechanisms behind low awareness, and could guide individual care planning.

Our previous study with a large sample of people with mild‐to‐moderate dementia found that a small proportion showed low awareness of their condition, and cross‐sectional associations of differing levels of awareness were explored.^18^ In this study, we follow up those people who appeared to lack awareness at baseline and aim to answer the following research questions: (1) Does awareness of condition in people with mild‐to‐moderate dementia change over time? (2) How do people who continue to show low awareness differ from those who subsequently demonstrate awareness?

## 2 METHODS

### 2.1 Study design

This study employs data collected in the Improving the experience of Dementia and Enhancing Active Life (IDEAL) cohort,^19^ a large, ongoing study of community‐dwelling people with mild‐to‐moderate dementia and their carers. The study presents a retrospectively matched observational case‐series exploring data from three timepoints.

Ethics approval for IDEAL was given by the Wales Research Ethics Committee 5 (reference 13/WA/0405) and the Ethics Committee of the School of Psychology, Bangor University (reference 2014‐11684). The programme was registered with UKCRN, registration number 16593. This study reports data from v5 of the IDEAL datasets.

### 2.2 Setting

IDEAL recruited people with mild‐to‐moderate dementia of any type from 29 UK National Health Service sites throughout England, Scotland, and Wales. Inclusion and exclusion requirements are described in detail elsewhere.^19^ These include a Mini‐Mental State Examination^20^ (MMSE) score of 15 or above at enrolment, equivalent to a Montreal Cognitive Assessment score of 8,^21^, ^22^ and capacity at baseline to give informed consent to take part. Baseline data were collected between 2014 and 2016. Willing participants were followed up 12 and 24 months later. There were 1537 participants with dementia in the IDEAL cohort at baseline. Previously we examined baseline data for 917 participants with complete data on an awareness measure.^18^ Here, we followed‐up those of the original 917 participants who remained in the programme at Timepoint 2 (T2) and Timepoint 3 (T3).

### 2.3 Participants

Among the sample of 917 people at baseline, there were two subgroups: people who demonstrated awareness of their condition and those who had no apparent awareness (‘low awareness’). Awareness status was re‐examined at T2 and T3. Reasons for withdrawal at T2 and T3 were recorded. Individuals who showed stable low awareness (SL) at all three timepoints were compared with those who had both gained awareness at T2 and retained awareness at T3. Cases for comparison were matched by age, sex, and dementia subtype, considering education, social class, and area‐level deprivation where possible.

### 2.4 Measures

#### 2.4.1 Awareness

Using the validated screening checklist of nine items from the Representations and Adjustment to Dementia Index^23^ (RADIX), self‐reports at each timepoint were categorised as either showing awareness or having low awareness of condition.^18^ People with dementia who endorsed one or more items were considered to show awareness whilst those who did not endorse any of the nine items were considered to have low awareness.

#### 2.4.2 Demographic information

At baseline ***:*** standard demographic information was recorded that is, age and age group (<65, 65–69, 70–74, 75–79, and 80+ years), sex, dementia subtype: Alzheimer's disease (AD), vascular dementia (VaD), mixed AD/VaD, frontotemporal dementia, Parkinson's disease dementia, dementia with Lewy bodies, unspecified/other dementia; time since diagnosis, education level (no qualification, school leaving certificate at age 16 years or at age 18 years, university), social class based on main lifetime occupation^24^ that is, I (Professional), II (Managerial and technical), III‐NM (Skilled non‐manual), III‐M (Skilled manual), IV (Partly skilled), V (Unskilled), and other categories ‘Armed forces’, ‘Not applicable’ and ‘Missing’. Area deprivation was categorised in quintiles from postcode information and nationally available data.^25^

#### 2.4.3 Cognitive tests

At each timepoint: MMSE^20^ was administered to demonstrate stage of dementia, in addition to its use as an inclusion criterion at baseline.

#### 2.4.4 Self‐report measures completed by the person with dementia

At baseline: Personality traits were assessed using the Mini‐International Personality Item Pool.^26^ Other psychological attributes were measured using self‐reported scales for optimism (Life‐Orientation Test‐Revised^27^ using six items without the filler items), self‐esteem (Rosenberg Self‐Esteem Scale^28^), and self‐efficacy (Generalized Self‐Efficacy Scale^29^). For each measure, higher scores indicate a higher level of that trait or attribute.

At all timepoints: Measures comprised the 10‐item Geriatric Depression Scale^30^ (GDS‐10), with higher scores indicating more depressed mood, and measures for perceived ability to live well, assessing QoL (Quality of Life in Alzheimer's Disease Scale^31^), satisfaction with life (Satisfaction with Life Scale^32^), and well‐being (World Health Organization‐Five Well‐being Index^33^). Perceived functional ability was rated by the person with dementia using the self‐reported modified 11‐item Functional Activity Questionnaire^4^, ^34^ (FAQ). Higher scores indicate poorer perceived functioning in everyday activities.

Subjective memory was also recorded at T2 and T3 using the question: ‘Compared to other people your age how would you describe your day‐to‐day memory?’ with a six‐point response scale ranging from excellent to very poor.^35^

#### 2.4.5 Informant measures completed by the carer

At all timepoints: the carer completed the informant‐rated FAQ, with higher scores indicating greater perceived impairment. Carers also recorded informant‐rated neuropsychiatric symptoms using the Neuropsychiatric Inventory Questionnaire^36^, ^37^ and the total symptom score was used. Carer stress was measured using the Relative Stress Scale^38^; higher scores indicate more stress resulting from the carer role.

#### 2.4.6 Co‐morbidity

At each timepoint ***:*** Using the Charlson Comorbidity Index^39^, ^40^ (CCI), symptoms related to medical conditions other than dementia, that affect mortality, and the type of condition, were jointly recorded by the person with dementia and carer at baseline, and by the carer as informant at subsequent timepoints. Where no carer was involved, the person with dementia self‐reported non‐dementia symptoms and comorbid conditions. The number of prescribed medications was recorded at each timepoint. For the carer, the total number of symptoms was recorded with self‐reported CCI. Subjective health was also self‐rated by the person with dementia and the carer using a single question,^41^ ‘Overall, how would you rate your health in the past 4 weeks?’ with a 6‐point Likert‐type response ranging from excellent to very poor.

### 2.5 Analyses

Using case comparisons, differences were explored between people with SL and people who gained awareness after T1. Matched cases were compared descriptively to investigate patterns of differences in other demographic details, cognition, functioning, mood, neuropsychiatric symptoms, psychological and personality variables, and co‐morbidity.

## 3 RESULTS

### 3.1 Dropout

At T1 there were 834 people showing awareness and 83 with low awareness. Of the original low awareness group, 61.4% had dropped out by T3 compared to 42.7% of the T1 group with awareness; see Table S1. The most frequent reason for withdrawal in both groups was health concerns. People from the low awareness group were also more likely to report lack of interest in the study or over‐commitment as reasons for withdrawing.

A description of the participants from the baseline low awareness group who remained in the study at T2 and T3 is shown in Table 1.

**TABLE 1:** Description of participants in the T1 low awareness group

Variable	T1 (*n* = 83)	T2 (*n* = 49)	T3 (*n* = 25)
Sex *n* (%)			
Male	50 (60.2)	33 (67.3)	15 (60.0)
Female	33 (39.8)	16 (32.7)	10 (40.0)
Age in years: Mean (SD) range	78.34 (9.14) 56–94	78.08 (8.37) 57–94	79.08 (7.65) 63–89
Dementia subtype *n* (%)			
Alzheimer's disease (AD)	47 (56.6)	28 (57.1)	18 (72.0)
Vascular dementia (VaD)	12 (14.5)	6 (12.2)	4 (16.0)
Mixed AD/VaD	13 (15.7)	10 (20.4)	3 (12.0)
Frontotemporal dementia	7 (8.4)	3 (6.1)	‐
Parkinson's disease dementia	1 (1.2)	1 (2.0)	‐
Dementia with Lewy bodies	1 (1.2)	1 (2.0)	‐
Unspecified/Other	2 (2.4)	‐	‐
Time since diagnosis (at T1) *n* (%)			
Less than 1 year	39 (47.0)	20 (40.8)	12 (48.0)
1–2 years	19 (22.9)	13 (26.5)	8 (32.0)
3–5 years	11 (13.3)	8 (16.3)	4 (16.0)
missing	14 (16.9)	8 (16.3)	1 (4.0)
Education *n* (%)			
No qualifications	22 (26.5)	12 (24.5)	7 (28.0)
School leaving certificate at age 16	16 (19.3)	8 (16.3)	5 (20.0)
School leaving certificate at age 18	28 (33.7)	19 (38.8)	9 (36.0)
University	15 (18.1)	10 (20.4)	4 (16.0)
missing	2 (2.4)	‐	‐
Area deprivation *n* (%)			
Quintile 1 most deprived	13 (15.7)	9 (18.4)	4 (16.0)
Quintile 2	13 (15.7)	7 (14.3)	4 (16.0)
Quintile 3	16 (19.3)	6 (12.2)	5 (20.0)
Quintile 4	19 (22.9)	16 (32.7)	6 (24.0)
Quintile 5 least deprived	22 (26.5)	11 (22.4)	6 (24.0)
Carer participating	67 (80.7)	41 (83.7)	20 (80.0)
Relationship with carer			
Spouse/partner n (%)	56 (83.6)	37 (90.2)	19 (95)
Other family/friend n (%)	11 (16.4)	4 (9.8)	1 (1)
MMSE mean (SD) range	22.26 (3.33) 16–29 missing 2	19.76 (4.85) 8–30	19.68 (4.58) 10–29
FAQ‐I mean (SD) range	20.69 (8.18) 0–33 missing 21	23.65 (8.29) 0–33 missing 9	24.35 (6.56) 10–33 missing 5
Awareness group			
Low awareness n (%)	83 (100)	21 (42.9)	10 (40)
Some awareness n (%)	0	28 (57.1)	15 (60)

Abbreviations: FAQ‐I, Functional Activities Questionnaire Informant rated; MMSE, Mini‐Mental State Examination.

### 3.2 Changes in awareness

From the T1 low awareness group, 28 people (33.7%) showed awareness at T2, and 15 people (18%) showed awareness at T3, with the number of checklist items endorsed at these timepoints ranging from one to nine. Details of these awareness subgroups are shown in Table S2. In contrast, there was a lesser extent of change in people who had initially showed awareness at T1 but had low awareness at T2 (40; 4.8%) and at T3 (32; 3.8%); see Figure S1.

**FIGURE 1:**
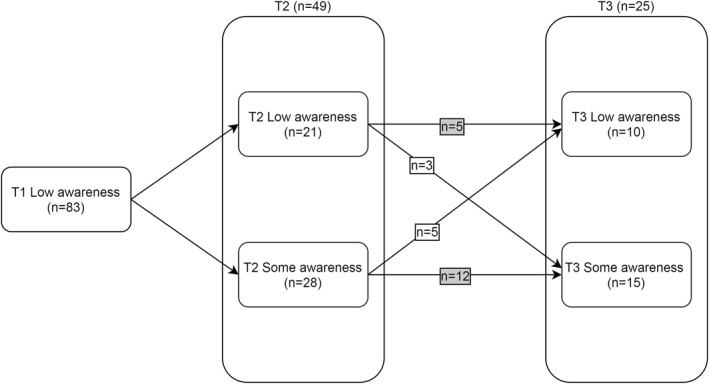
Changes from low awareness at T1. Highlighted boxes indicate cases with stable low awareness (*n* = 5) and those with persistent gains in awareness (*n* = 12)

A small number of people (n = 5) were stable in exhibiting low awareness that is, endorsing no checklist items at any timepoint. Twelve people, having gained awareness at T2, showed persisting gains at T3; see Figure 1.

### 3.3 Case comparisons

The five SL were matched with cases showing persistent gains in awareness (PG) as described above. For further details of case matching see Table S3. Case comparisons are summarised in Table 2, with additional data available in Tables S4a‐f.

**TABLE 2:** Summary of case comparisons

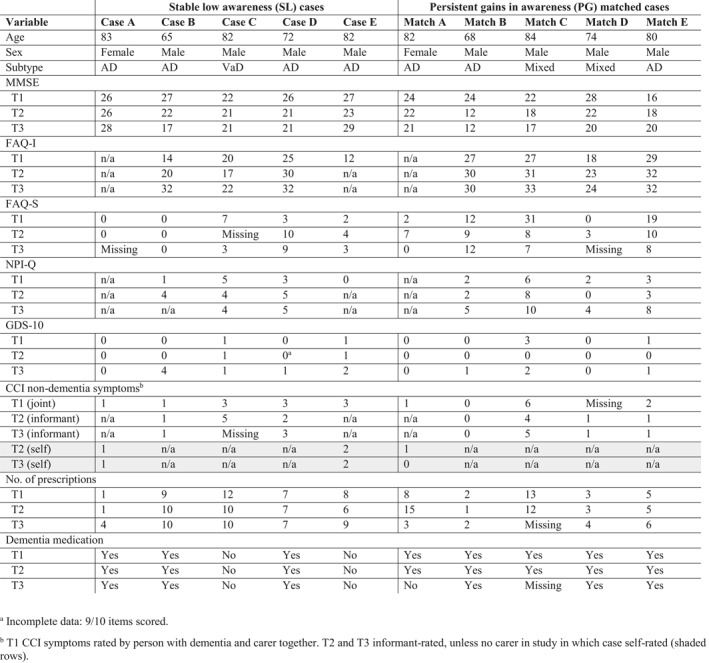

Abbreviations: AD, Alzheimer's disease; CCI, Charlson Comorbidity Index; FAQ‐I, Functional Activities Questionnaire informant rated; FAQ‐S, Functional Activities Questionnaire self‐rated; GDS‐10, Geriatric Depression Scale‐10; Mixed, mixed Alzheimer's disease and vascular dementia; MMSE, Mini‐Mental State Examination; NPI‐Q, Neuropsychiatric Inventory Questionnaire total symptoms; VaD, vascular dementia.

### 3.4 Demographic characteristics

Of the five SL cases, four were men, and four had AD. There was no consistent pattern for age, education, or socio‐economic status. None lived in deprived areas. One person was living in residential care by T3; see Table S4a.

There was no clear difference in time since diagnosis between the cases and matches at T1. At T1 and T2 most people showing awareness were within 2 years of diagnosis, but there were later awareness gains, up to four or more years after diagnosis; see Table S5.

### 3.5 Cognition

**FIGURE 2:**
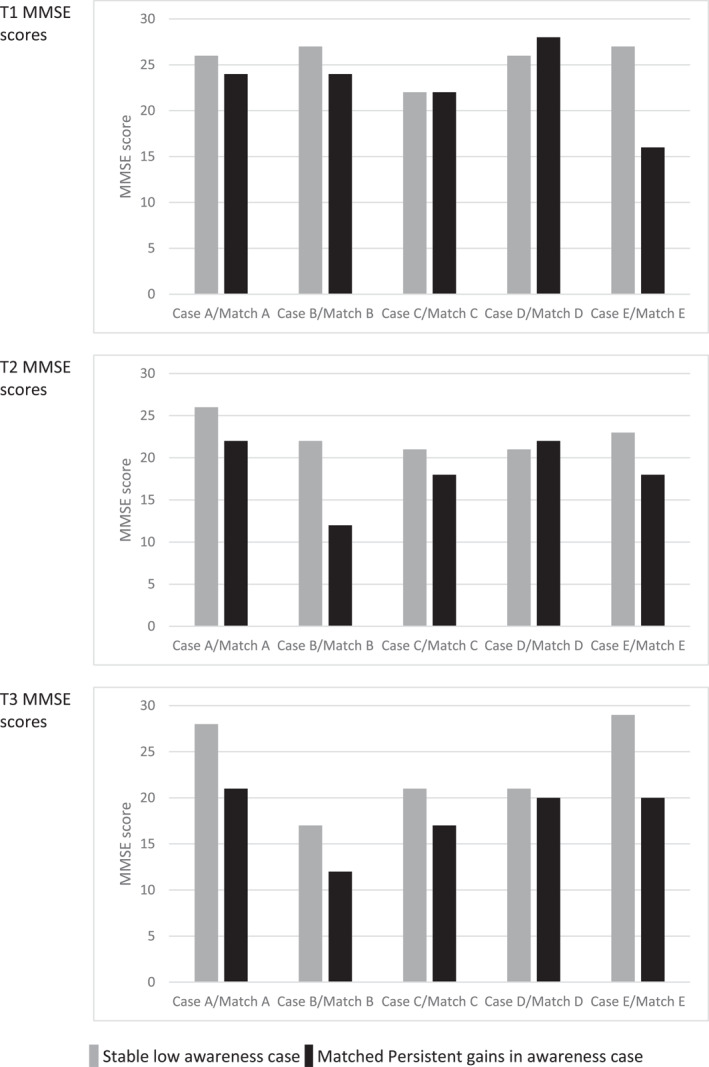
Mini‐Mental State Examination (MMSE) scores at T1, T2, and T3

Mini‐Mental State Examination scores indicated mild dementia in the SL cases at all timepoints, excepting Case B who scored 17 at T3. In comparison, the matched PG cases mainly had lower MMSE scores at each timepoint; three had scores <20 at either T2 or T3, see Table 2 and Figure 2. No clear differences were seen for self‐rated memory; see Table S4b.

### 3.6 Functional ability

**FIGURE 3:**
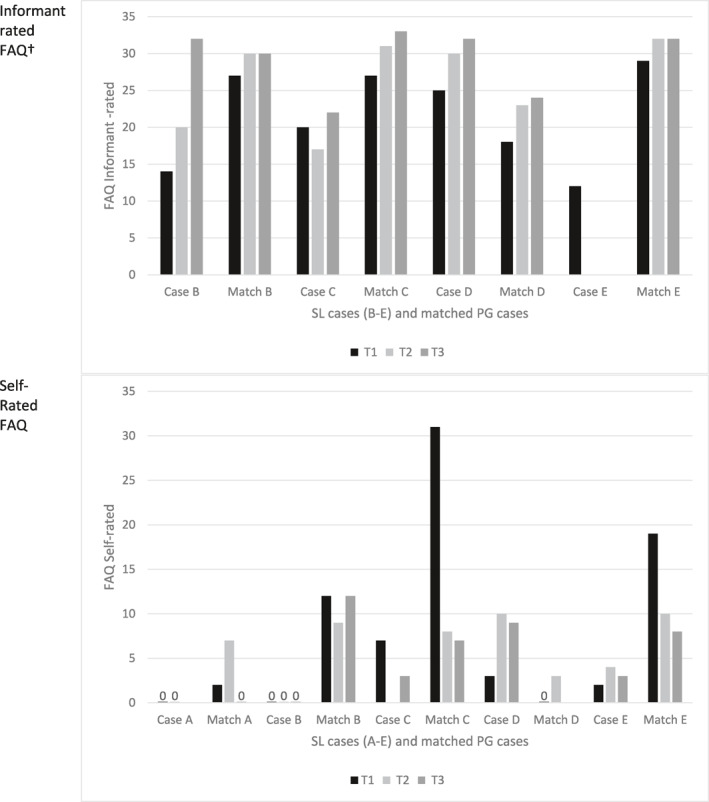
Functional Activities Questionnaire scores rated by self and informant at T1, T2, and T3. † Case A and Match A had no informant at any timepoint. Case E had no informant at T2 and T3. FAQ, Functional Activities Questionnaire; PG, persistent gains in awareness; SL, stable low awareness

The self‐rated FAQ scores reflect better perceived function in activities of daily living in the SL cases compared to the PG cases, with the exception of Case D who perceived more difficulties. Where a carer was available, informant ratings indicated greater functional difficulties in the PG cases, apart from apparent deterioration in Case B at T3, and Case D who was rated more impaired at all timepoints than the matched case. See Table 2 and Figure 3.

### 3.7 Psychiatric and psychological variables

There was no obvious pattern of difference in depression scores or neuropsychiatric symptoms between the groups; see Table 2. Similarly, there were no obvious differences in self‐rated personality and psychological traits; see Table S4c.

### 3.8 Co‐morbidity

Three of the five SL cases had more non‐dementia symptoms and more prescriptions than their matched cases; see Table 2 and Figure S2. Symptoms were self‐reported at one or more timepoint where no informant was available for two of the SL cases, and one of the PG cases. Dementia medication was prescribed for three of the SL cases, compared to all five of the PG cases. There was no clear difference in self‐rated health or the type of co‐morbid conditions between the groups; see Tables S4d and S6.

### 3.9 Living well

**FIGURE 4:**
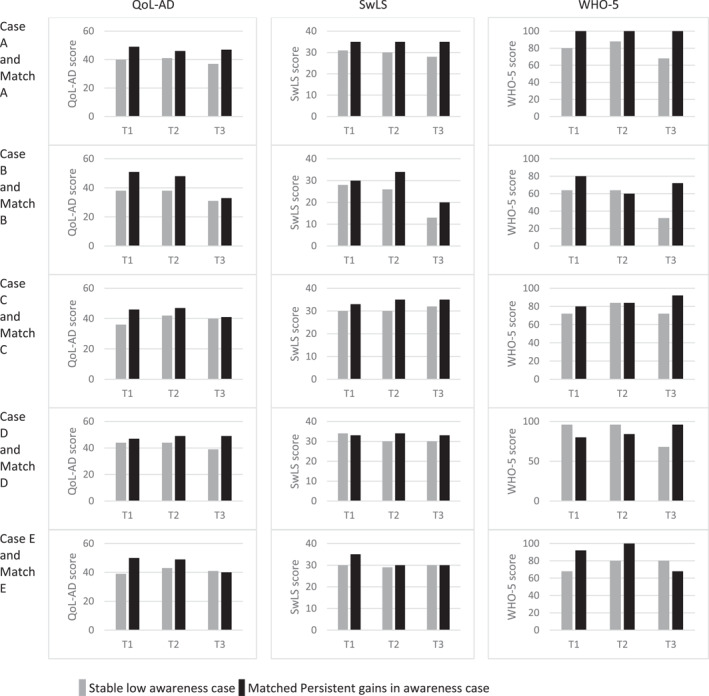
Scores on measures of ‘living well’ at T1, T2, and T3. QoL‐AD, Quality of Life Alzheimer's Disease Scale; SwLS, Satisfaction with Life Scale; WHO‐5, World Health Organization‐Five Well‐being Index percentage score

A clear difference was seen in scores for QoL, life satisfaction, and well‐being between the groups, with generally lower scores for the SL cases; see Figure 4 and Table S4e.

### 3.10 Carer co‐morbidity and stress

No clear pattern was seen in comparing carer co‐morbid symptoms, self‐rated health, or stress; see Table S4f.

## 4 DISCUSSION

This study is one of few that have explored change in awareness of condition in people with mild‐to‐moderate dementia over time. Over a 2‐year period, changes in awareness were seen in both directions and, even allowing for greater attrition, gains in people with low awareness at baseline were more likely than declines in those who initially showed awareness. There were gains in expressed awareness as late as four or more years after diagnosis. Matched case comparisons helped investigate descriptive differences between people who acquired awareness and those with SL. Few patterns of differences were evident between these groups. Although heterogeneous, the people with ongoing low awareness generally had equal or better cognitive and perceived functional ability, and slightly more co‐morbidity, than people who developed some awareness.

We found that awareness could improve despite cognitive decline, consistent with a study of people with similar baseline cognitive scores.^8^ Results diverge from studies that included people with more impaired cognition, where worsening cognitive function^6^ or deteriorating informant‐rated functional ability^7^ were features of ongoing or newly impaired awareness. In contrast, we found that the SL cases had better self‐ and informant‐reported functional ability. This suggests that different mechanisms behind low awareness may be important in people with mild dementia.

Evidence is lacking concerning changes in awareness in relation to time since diagnosis, with findings elsewhere mostly from cross‐sectional studies,^42^ showing either no association, or that longer duration of illness was associated with lower awareness. One study found that awareness of memory performance on a metacognitive task declined before diagnosis, and was typically impaired by the time of diagnosis.^43^ In our sample, most people demonstrated some awareness of their difficulties within 2 years of diagnosis but some first expressed awareness on our screening measure much later, four or more years after diagnosis.

The reported co‐morbidity among the SL group may suggest a significant non‐dementia disease burden, although this was not reflected in self‐rated health, and the group differences were slight. Prioritisation of physical health symptoms has been reported in people with undiagnosed dementia, with a tendency toward normalising symptoms of cognitive decline.^44^ Symptoms of dementia are often not viewed as ‘illness’^45^, ^46^ and may have seemed less important, particularly if co‐morbid conditions and treatment were well‐established before the onset of dementia. This merits further investigation. Medication for dementia was prescribed for all five PG cases, but their gains in awareness were not associated with cognitive or functional improvements. It is unclear whether changes were related to dementia medication, which was also prescribed to three of the five SL cases. No significant association between use of acetylcholinesterase inhibitor medication and awareness was seen in an older study.^47^ Awareness is infrequently assessed formally in clinical situations, or investigated in relation to dementia medication. This would be an important addition when assessing outcomes from dementia medication.

Other psychiatric conditions in people with dementia can complicate the assessment of awareness. For example, a person with delusional ideas due to psychosis could display an unrealistic understanding of their situation or abilities. Psychosis and other neuropsychiatric symptoms can co‐exist with impaired awareness, particularly in more severe dementia, but are clinically distinguishable and correlations are inconsistent.^48^ Here, the SL cases had a low reported number of neuropsychiatric symptoms, as also seen in the overall cohort.^18^ Alternatively, depression is known to influence awareness in dementia.^10^ People with depression are often more aware of their difficulties^49^ and pessimistic about their abilities, and lower awareness of dementia has been associated with less depressed mood.^18^ However, in our case comparisons, the 10 cases generally had low GDS‐10 scores indicating mood was not depressed.

Although no clear differences were seen in personality traits or positive psychological attributes, the PG cases maintained better scores for QoL, life‐satisfaction and wellbeing, before and after awareness gains were shown. This is surprising, and contrasts with lower QoL scores in the group who showed awareness at baseline.^18^ Conclusions on whether awareness relates positively or negatively to QoL remain mixed.^50^ Perhaps what matters are the circumstances that increase awareness, and whether there is sufficient support in place when there is awareness of dementia symptoms and diagnosis.

### 4.1 Strengths

The study examines longitudinal data at three timepoints from a large sample at baseline. Access to a range of variables enabled a detailed look at people with different awareness profiles, categorised by self‐report with a screening checklist, which demonstrated good reliability in a validation study.^23^

### 4.2 Limitations

Due to attrition, the subgroups for comparison at T3 were small. The use of matched cases served to accommodate this to some extent but the difficulty of drawing conclusions based on five cases is recognised. There was a low threshold for judging the presence of awareness: acknowledging a minimum of one difficulty on the screening checklist. However, people with awareness gains endorsed between one and nine items at later timepoints, indicating authentic change. The checklist^23^ does not purport to measure awareness across all domains of everyday functioning. Considering the heterogeneity of awareness,^11^ the findings may not extend beyond awareness of condition.

The participants remaining in the study at T3 may have been more aware than those who dropped out, indicated by their willingness to take part in dementia research, or influenced by repeated visits from researchers investigating dementia. Some may have held more awareness than they expressed, or were willing to disclose to a researcher. This would be difficult to determine; commonly, studies rely on self‐evaluation of abilities, often in comparison to informant‐ratings. Otherwise, when little awareness of difficulties is expressed verbally, a degree of implicit awareness can sometimes be inferred through observation of non‐verbal responses or self‐adjustments to activities.^51^ This implicit awareness appears to accommodate changes in ability or condition.^52^, ^53^ However, few studies have assessed implicit awareness.^5^

Recording of non‐dementia symptoms relied on self‐report at one or more timepoint for two of the SL cases and only one of the PG cases. Confirmation with medical records was not possible to determine accuracy of this information. Issues around accuracy of self‐reporting have been discussed in a previous paper,^54^ with literature indicating that under‐reporting is more common than over‐reporting when compared to medical records.^55^, ^56^ The self‐reported number of symptoms in these cases are low, and any impact of self‐reporting on results is unclear.

We do not know what information was provided to people between timepoints,^57^ if any, and whether awareness gains were related to better information provision, more opportunities to discuss dementia, a critical event, or greater assimilation of existing facts and experiences leading to more accurate self‐knowledge.^58^ Despite access to a range of variables there may have been other important differences between individuals and their life experiences that would only be apparent through qualitative interviews.

### 4.3 Implications

Our results should be interpreted with caution due to the very small numbers in the groups of interest at T3. However, the fact that only five people continued to show low awareness throughout is noteworthy, and contrasts with the greater number who gained awareness during the study period, allowing some tentative conclusions to be drawn.

The results show the dynamic nature of awareness. In mild‐to‐moderate dementia, there is no simple relationship between changes in apparent awareness and the degree of cognitive or functional difficulties. There may be different reasons for ongoing low awareness in an individual, which are not entirely clear but reach beyond the stage of disease. Initial lack of awareness may represent a reaction to the diagnosis or a coping response, rather than a symptom of the condition.^13^ Awareness may increase over time, particularly as more difficulties are encountered in everyday life. Declining function and the experience of struggling with everyday activities, prompting feedback from others and greater need for assistance, may trigger greater acknowledgement.^4^ Conversely, persevering and continuing to manage, or lack of feedback from others, may delay awareness.

Research challenges around measuring awareness are well‐recognised.^5^, ^42^ In many studies, for example, investigating the correlates of impaired awareness, awareness is measured with the assumption that low awareness is fixed or progressively worsening. This is not always the case for people with mild‐to‐moderate dementia. A single assessment in a cross‐sectional study, even using a highly reliable and valid measure, could produce misleading results in individuals who subsequently develop awareness. There should be ongoing consideration of the non‐biological factors influencing awareness^3^ and an understanding that expressed awareness may change. It is important for clinicians to recognise that individuals may understate their difficulties, at least for a time, but, alongside their carers, they may require different types of support as awareness of difficulties develops. Co‐morbid conditions may detract from acknowledgement of dementia, perhaps with difficulties attributed to physical disease rather than cognitive impairment, but it remains important to seek the priorities of the person with dementia.^59^ Appropriate support may enable maintenance of good QoL as awareness emerges, which could be several years after disclosure of the dementia diagnosis.

## 5 CONCLUSIONS

Awareness of the dementia condition can change over time. Low awareness in people with mild‐to‐moderate dementia is not simply a function of cognitive or functional difficulties. Awareness remains a complex phenomenon, not entirely explained by disease stage. Gains in awareness may occur several years after a dementia diagnosis and clinicians and researchers should be prepared to recognise this and respond accordingly.

## CONFLICT OF INTEREST

The authors declare that they have no conflict of interest.

## ETHICS APPROVAL STATEMENT

Ethics approval for IDEAL was given by the Wales Research Ethics Committee 5 (reference 13/WA/0405) and the Ethics Committee of the School of Psychology, Bangor University (reference 2014‐11684). The programme was registered with UKCRN, registration number 16593.

## PARTICIPANT CONSENT STATEMENT

All participants provided informed consent to take part in the study at baseline.

## AUTHOR CONTRIBUTIONS

Catherine M. Alexander analysed and interpreted the data, and prepared the draft manuscript. Anthony Martyr helped design the study and contributed to writing the manuscript. Linda Clare conceived and designed the idea for the study and contributed to writing the manuscript. All authors reviewed and approved the final manuscript.

## Supporting information

Supplementary Material S1

## Data Availability

IDEAL data were deposited with the UK data archive in April 2020 and will be available to access from April 2023. Details of how the data can be accessed after that date can be found here: https://reshare.ukdataservice.ac.uk/854293/. This study reports data from v5 of the IDEAL datasets.
