# Supplementary material for: Changes in awareness of condition in people with mild‐to‐moderate dementia: Longitudinal findings from the IDEAL cohort
Source: Int J Geriatr Psychiatry. 2022 Mar 16;37(4):10.1002/gps.5702. doi: 10.1002/gps.5702 (PMC9314100; doi:10.1002/gps.5702)

Supplementary Table 1. Reasons for drop-out from the study.

T1 Awareness group	Status	Drop-out at T2<br>n (% of T1 group)	Drop-out at T3<br>n (% of T1 group)
T1 Low awareness group (n=83)	Withdrew: died	3 (3.6)	5 (6.0)
	Withdrew: health	8 (9.6)	7 (8.4)
	Withdrew: carer health	2 (2.4)	-
	Withdrew: couldn't cope/too challenging	-	2 (2.4)
	Withdrew: too much commitment	5 (6.0)	2 (2.4)
	Withdrew: not interested	5 (6.0)	1 (1.2)
	Withdrew: too distressing	1 (1.2)	-
	Lost to follow-up	7 (8.4)	3 (3.6)
	Total withdrew at time-point	31 (37.3)	20 (24.1)
T1 Some Awareness (n=834)	Withdrew: died	29 (3.5)	36 (4.2)
	Withdrew: health	50 (6.0)	59 (7.1)
	Withdrew: carer health	4 (0.5)	3 (0.4)
	Withdrew: couldn't cope/too challenging	6 (0.7)	10 (1.2)
	Withdrew: too much commitment	16 (1.9)	13 (1.6)
	Withdrew: not interested	24 (2.9)	10 (1.2)
	Withdrew: too distressing	2 (0.2)	4 (0.5)
	Withdrew: bereavement	3 (0.4)	1 (0.1)
	Withdrew: moved to care home	1 (0.1)	1 (0.1)
	Participant withdrew: moved out of area and could not be contacted	1 (0.1)	1 (0.1)
	Withdrew: lost to follow-up	41 (4.9)	34 (4.1)
	Didn't participate in this time-point only	7 (0.8)	-
	Total withdrew at time-point	184 (22.1)	172 (20.6)

T1 timepoint 1; T2 timepoint 2; T3 timepoint 3

Supplementary Table 2. Baseline low awareness group: Awareness subgroups at T2 and T3

	T2		T3	
	**Low awareness (n=21)**	**Some awareness (n=28)**	**Low awareness (n=10)**	**Some awareness (n=15)**
**Age** mean (SD) range	79.38 (7.03)<br>66-89	77.11 (9.25)<br>57-94	82.30 (6.50)<br>67-89	76.93 (7.80)<br>63-86
**Sex**				
Male	13 (61.9)	20 (71.4)	7 (70.0)	8 (53.3)
Female	8 (38.1)	8 (28.6)	3 (30.0)	7 (46.7)
**Subtype**				
AD	15 (71.4)	13 (46.4)	8 (80.0)	10 (66.7)
VaD	2 (9.5)	4 (14.3)	2 (20.0)	2 (13.3)
Mixed AD/VaD	3 (14.3)	7 (25.0)	-	3 (20.0)
Other	1 (4.8)	4 ((14.3)	-	-
**MMSE** mean (SD) range	19.33 (5.35)<br>8-30	20.07 (4.51)<br>9-26	20.40 (4.79)<br>15-29	19.20 (4.54)<br>10-27
**FAQ-I** mean (SD) range	23.69 (8.29)<br>0-30<br>missing 5	23.63 (8.46)<br>3-33<br>missing 4	25.50 (4.47)<br>20-32<br>missing 2	23.58 (7.74)<br>10-33<br>missing 3
**NPI-Q** mean (SD) range	4.18 (2.65)<br>0-9<br>missing 4	3.92 (2.64)<br>0-10<br>missing 4	3.86 (1.22)<br>2-5<br>missing 3	4.55 (2.91)<br>0-10<br>missing 4
**Depressed mood** n (%)	0 (0)	5 (18.5) missing 1	1 (10)	0 (0)

Depressed mood recorded if Geriatric Depression Scale-10 score ≥ 4.

Abbreviations: AD Alzheimer's disease; VaD vascular dementia; Other includes Frontotemporal dementia, Dementia with Lewy bodies, Parkinson's disease dementia, and Unspecified/other dementia; MMSE: Mini-Mental State Examination; FAQ-I Functional Activities Questionnaire informant rated; NPI-Q Neuropsychiatric Inventory Questionnaire total symptoms.

Supplementary Table 3. Details of case matching.

Cases A-E are the stable low awareness group. Cases 1-12 are the persistent gains in awareness group.

	Sex	Age group (y)	Subtype	Education	*Social Class ONS*	*Area deprivation quintile*	Reasoning
**Case A**	**Female**	**80+**	**AD**	**School leaving certificate age 16**	***III-NM***	***Q5 least deprived***	
Case 2= Match A	✓	✓	✓	School leaving certificate age 18	*III-M*	*Q4*	Good match
Case 1	✓	75-79	✓	No qualification	*N/A*	*Q3*	Not matched on age
Case 4	✓	65-69	✓	University	*N/A*	*Q3*	Not matched on age
Case 8	✓	75-79	✓	School leaving certificate age 18	✓	✓	Not matched on age, otherwise reasonable match.
Case 10	✓	65-69	VaD	✓	✓	*Q2*	Not matched age and subtype.
**Case B**	**Male**	**65-69**	**AD**	**University**	***IV***	***Q4***	
Case 5= Match B	✓	✓	✓	✓	*I*	*Q5 least deprived*	Good match
**Case C**	**Male**	**80+**	**VaD**	**No qualification**	***IV***	***Q5 least deprived***	
Case 7= Match C	✓	✓	Mixed AD/VaD	School leaving certificate age 18	*III-NM*	*Q2*	Closest match for first 3 variables
Case 9	✓	65-69	Mixed AD/VaD	School leaving certificate age 18	*II*	*Q3*	Different age group, not closely matched on any variables other than gender.

Case 11	✓	✓	AD	✓	*III-M*	*Q1 most deprived*	Different subtype, greater difference in deprivation
Case 12	✓	<65	✓	✓	*II*	*Q1 most deprived*	Different age group, greater difference in deprivation.
**Case D**	**Male**	**70-74**	**AD**	**School leaving certificate age 16**	***III-NM***	***Q4***	
Case 6= Match D	✓	✓	Mixed AD/VaD	School leaving certificate age 18	✓	*Q3*	Best match for age and sex.
Case 11	✓	80+	✓	No qualification	*III-M*	*Q1 most deprived*	Different age group, greater deprivation.
**Case E**	**Male**	**80+**	**AD**	**University**	***II***	***Q4***	
Case 3= Match E	✓	✓	✓	No qualification	*III-NM*	*Q5 least deprived*	Best match for age, sex, subtype (and SC and deprivation) but education not matched
Case 7	✓	✓	Mixed AD/VaD	School leaving certificate age 18	*III-NM*	*Q2*	Different subtype.
Case 11	✓	✓	✓	No qualification	*III-M*	*Q1 most deprived*	Similar to case 3 but less well matched for SC and area deprivation.

Matched prioritizing sex/age group/dementia subtype then education. Social class and area deprivation considered for imperfect initial matches or where alternatives were equally matched. ✓ indicates matched category. Highlighted cases selected as matches.

Abbreviations: AD Alzheimer’s disease; VaD vascular dementia; ONS Office for National Statistics 2010 social classes: I (Professional), II (Managerial and technical), III-NM (Skilled non-manual), III-M (Skilled manual), IV (Partly skilled), V (Unskilled), N/A (not applicable).

Supplementary Tables 4a-f. Case comparisons for Stable Low awareness cases (Cases A-E) and matched cases from Persistent Gains in awareness group (Matches A-E).

4a) Demographic variables at T1

Case or Match	Age at T1 (y)	Sex	Dementia subtype	Time since diagnosis at T1	Education	Social Class ONS 2010	Area deprivation quintile	Relationship with carer	Change in carer during study
**Case A**	83	Female	AD	Less than 1 year	School leaving certificate at age 16	III-NM	Q5 least deprived	n/a	No carer in study T1-T3
**Match A**	82	Female	AD	Less than 1 year	School leaving certificate at age 18	III-M	Q4	n/a	No carer in study T1-T3
**Case B**	65	Male	AD	1-2 years	University	IV	Q4	Spouse	In care at T3
**Match B**	68	Male	AD	missing	University	I	Q5 least deprived	Spouse	No change in care
**Case C**	82	Male	VaD	Less than 1 year	No qualifications	IV	Q5 least deprived	Spouse	No change in care
**Match C**	84	Male	Mixed AD/VaD	Less than 1 year	School leaving certificate at age 18	III-NM	Q2	Spouse	No change in care
**Case D**	72	Male	AD	1-2 years	School leaving certificate at age 16	III-NM	Q4	Spouse	No change in care
**Match D**	74	Male	Mixed AD/VaD	Less than 1 year	School leaving certificate at age 18	III-NM	Q3	Spouse	No change in care
**Case E**	82	Male	AD	1-2 years	University	II	Q4	Spouse	Carer withdrew T2 onwards.
**Match E**	80	Male	AD	3-5 years	No qualifications	III-NM	Q5 least deprived	Spouse	No change in care

Abbreviations: AD Alzheimer's disease; VaD Vascular dementia; ONS Office for National Statistics 2010 social classes: I (Professional), II (Managerial and technical), III-NM (Skilled non-manual), III-M (Skilled manual), IV (Partly skilled), V (Unskilled)

4b) Person with dementia self-rated memory (SRM) at T2, T3		
**Case or Match**	**SRM T2**	**SRM T3**
**Case A**	Good	Good
**Match A**	Fair	Very good
**Case B**	Good	Good
**Match B**	Good	Fair
**Case C**	Fair	Fair
**Match C**	Fair	Fair
**Case D**	Very good	Good
**Match D**	Fair	Good
**Case E**	Good	Good
**Match E**	Good	Good

4c) Self-rated psychological and personality variables at T1								
**Case or Match**	**Optimism**	**Self-efficacy**	**Self-esteem**	**Agreeableness**	**Conscientiousness**	**Extraversion**	**Openness**	**Neuroticism**
**Case A**	14	38	32	15	14	16	16	9
**Match A**	20	40	40	19	8	15	17	4
**Case B**	20	26	31	18	14	9	10	7
**Match B**	18	35	30	19	18	17	14	8
**Case C**	14	30	missing	12	14	12	13	10
**Match C**	15	30	30	18	17	14	19	4
**Case D**	19	36	39	18	16	14	18	5
**Match D**	19	35	37	15	15	14	18	6
**Case E**	16	35	30	15	15	11	16	8
**Match E**	22	36	39	19	17	11	17	5

4d) Person with dementia self-rated health (SRH) at T1, T2, T3			
**Case or Match**	**SRH T1**	**SRH T2**	**SRH T3**
**Case A**	Very good	Good	Good
**Match A**	Good	Good	Very good
**Case B**	Fair	Very good	Good
**Match B**	Excellent	Good	Very good
**Case C**	Good	Very good	Very good
**Match C**	Good	Very good	Fair
**Case D**	Good	Very good	Good
**Match D**	Excellent	Excellent	Excellent
**Case E**	Very good	Excellent	Good
**Match E**	Very good	Excellent	Very good

4e) Living well at T1, T2, T3									
**Case or Match**	**Quality of Life in Alzheimer's Disease Scale**			**Satisfaction with Life Scale**			**World Health Organization-Five Well-being Index†**		
	**T1**	**T2**	**T3**	**T1**	**T2**	**T3**	**T1**	**T2**	**T3**
**Case A**	40	41	37	31	30	28	80	88	68
**Match A**	49	46	47	35	35	35	100	100	100
**Case B**	38	38	31	28	26	13	64	64	32
**Match B**	51	48	33	30	34	20	80	60	72
**Case C**	36	42	40	30	30	32	72	84	72
**Match C**	46	47	41	33	35	35	80	84	92
**Case D**	44	44	39	34	30	30	96	96	68
**Match D**	47	49	49	33	34	33	80	84	96
**Case E**	39	43	41	30	29	30	68	80	80
**Match E**	50	49	40	35	30	30	92	100	68

†percentage score.

4f) Carer self-rated Charlson Comorbidity Index (CCI) number of symptoms, self-rated health (SRH), and Relative Stress Scale (RSS) score at T1, T2, T3									
	**Carer CCI no. symptoms**			**Carer SRH**			**Carer RSS total**		
**Case or Match**	**T1**	**T2**	**T3**	**T1**	**T2**	**T3**	**T1**	**T2**	**T3**
**Case A**	n/a	n/a	n/a	n/a	n/a	n/a	n/a	n/a	n/a
**Match A**	n/a	n/a	n/a	n/a	n/a	n/a	n/a	n/a	n/a
**Case B**	4	2	2	Very good	Very good	Very good	9	9	7
**Match B**	0	0	0	Excellent	Excellent	Excellent	18	21	23
**Case C**	1	2	2	Good	Fair	Fair	23	24	24
**Match C**	2	3	missing	Fair	Fair	Fair	36	42	48
**Case D**	3	0	2	Good	Fair	Fair	37	33	34
**Match D**	2	0	0	Very good	Very good	Excellent	8	8	6
**Case E**	1	n/a	n/a	Very good	n/a	n/a	26	n/a	n/a
**Match E**	0	0	0	Good	Very good	Fair	22	30	33

Supplementary Table 5. Time since diagnosis when awareness shown. Highlighted cells indicate time-point when awareness shown.

	**Awareness at T1 (n=834)**	**Awareness gains at T2 (n=28)**	**Persistent Gains matched cases (n=5)**	**Awareness gains at T3 (n=3)**	**Stable low awareness (n=5)**
**Time since diagnosis at T1**					
Less than 1 year	440 (52.8)	16 (57.1)	3 (60)	-	2 (40)
1-2 years	245 (29.4)	5 (17.9)	-	2 (66.7)	3 (60)
3-5 years	87 (10.4)	5 (17.9)	1 (20)	1 (33.3)	-
6+ years	10 (1.2)	-	-	-	-
Missing	52 (6.2)	2 (7.1)	1 (20)	0	0
**Time since diagnosis at T2 (1 year after baseline)**					
1-2 years		16 (57.1)	3 (60)	-	2 (40)
2-3 years		5 (17.9)	-	2 (66.7)	3 (60)
4-6 years		5 (17.9)	1 (20)	1 (33.3)	-
7+ years		-	-	-	-
Missing		2 (7.1)	1 (20)	0	0
**Time since diagnosis at T3 (2 years after baseline)**					
2-3 years				-	2 (40)
3-4 years				2 (66.7)	3 (60)
5-7 years				1 (33.3)	-
8+ years				-	-
Missing				0	0

Supplementary Table 6. Co-morbid conditions recorded jointly at T1, and by informant at T2, T3.

	**Case A**			**Match A**			**Case B**			**Match B**			**Case C**			**Match C**			**Case D**			**Match D**			**Case E**			**Match E**		
	T1	T2	T3	T1	T2	T3 †	T1	T2	T3	T1	T2	T3	T1	T2	T3	T1	T2	T3	T1	T2	T3	T1	T2	T3	T1	T2	T3	T1	T2	T3
**MI**							Y									Y														
**CCF**					Y†											Y	Y	Y									Y†	Y		
**Hypertension**								Y	Y				Y	Y	Y	Y		Y	Y	Y	Y		Y	Y	Y	Y†	Y†	Y	Y	Y
**Depression**													Y	Y					Y		Y									
**PVD**		Y†	Y†													Y		Y							Y					
**Aortic aneurysm**																Y		Y												
**Poor circulation**		Y†	Y†																						Y					
**Cerebrovasc disease**				Y†												Y		Y												
**TIA**				Y†												Y														
**Chronic bad chest**														Y																
**Joint inflammation**	Y†													Y																
**RA**	Y†																													
**Skin ulcer**																Y		Y												
**Bedsores**																Y		Y												
**Cancer within last 5 years**													Y‡	Y‡	Y‡							Y‡								

† self-rated by person with dementia as no carer/informant available; ‡ prostatic cancer

Abbreviations. MI Myocardial infarction; CCF Congestive cardiac failure; PVD Peripheral vascular disease; Cerebrovasc Cerebrovascular; TIA Transient ischaemic attack; RA Rheumatoid arthritis.

Supplementary Figure 1. Changes in awareness from T1 to T3 with numbers who withdrew (w/d).

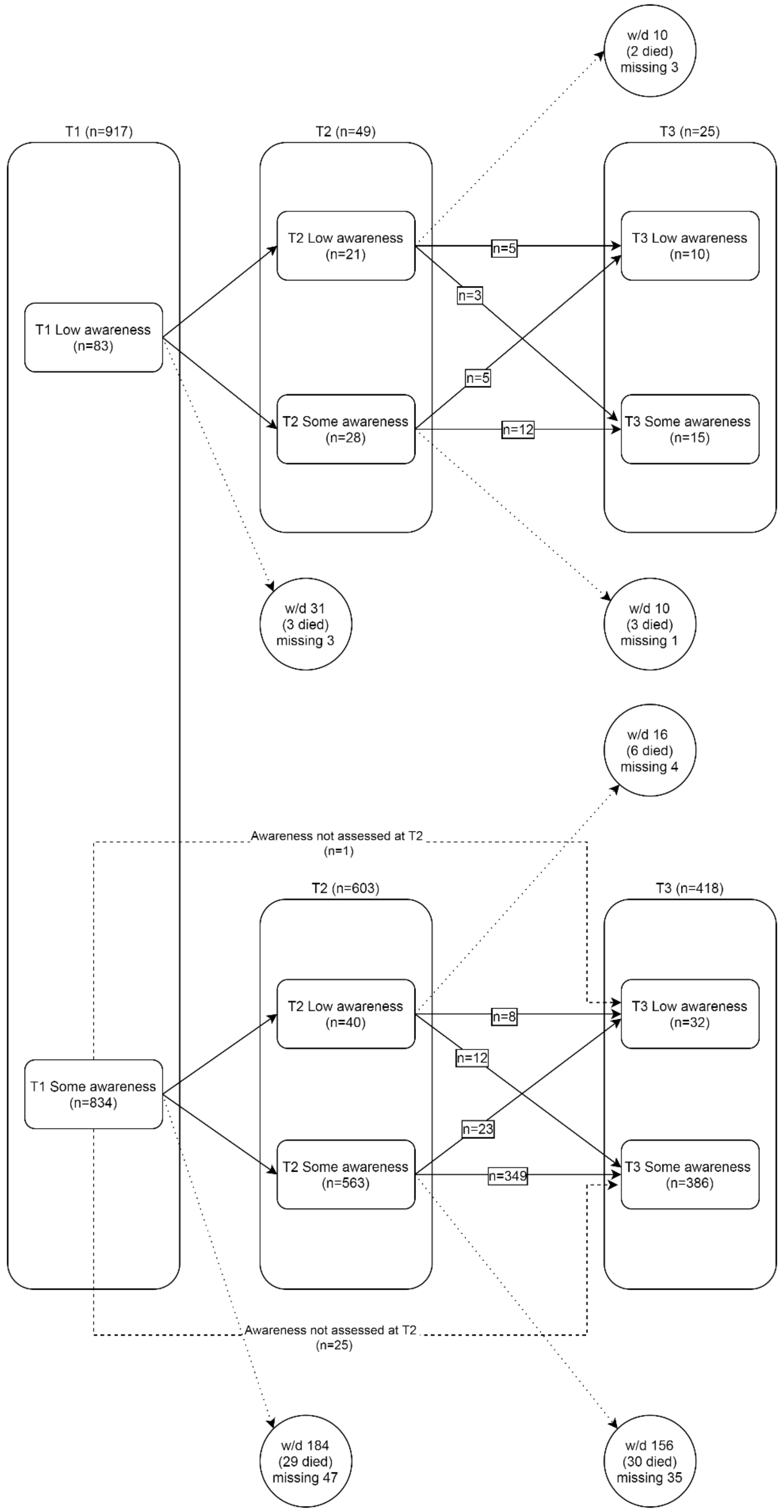

Supplementary Figure 2. Number of prescriptions and non-dementia symptoms in matched cases.

Number of prescriptions for SL cases and matched PG cases.

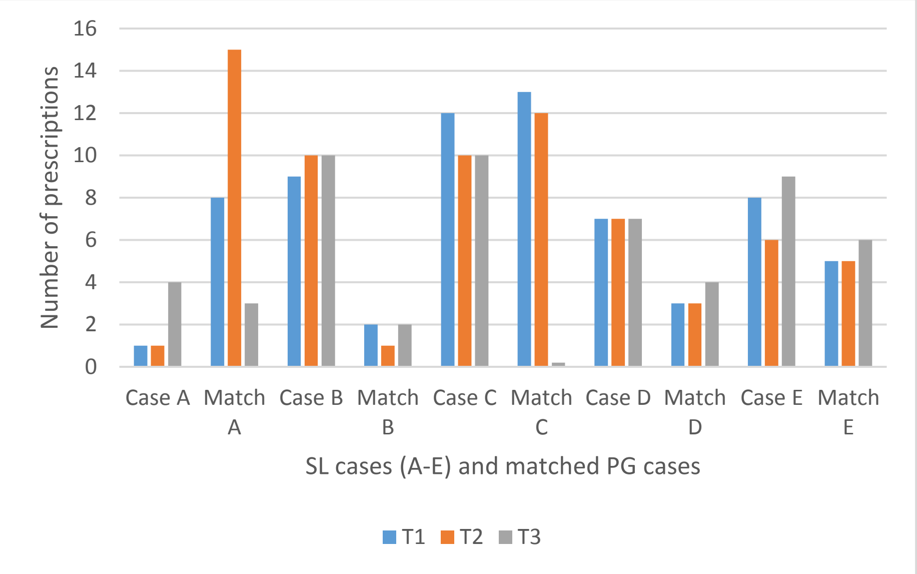

Number of non-dementia symptoms reported on Charlson Comorbidity Index (CCI)

Joint ratings at T1, informant-rated symptoms at T2 and T3 unless no informant available; self-rating was provided for Case A, Match A and Case E at T2 and T3.

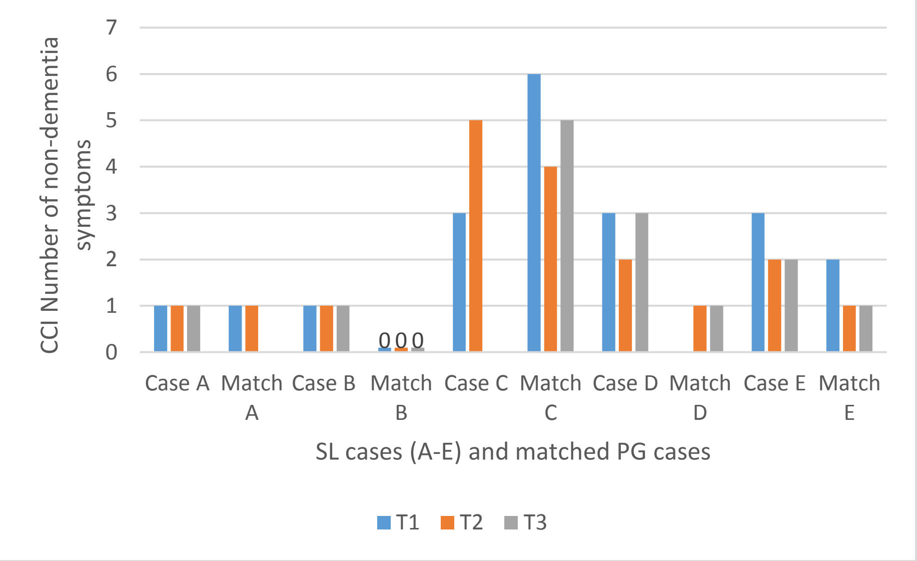

Supplement: Supplementary file 1 — Supplementary Material S1 [file GPS-37-0-s001.pdf]
